# Diverse effects of coexpression of human SOD1 variants on motor neuron disease

**DOI:** 10.1093/hmg/ddaf088

**Published:** 2025-06-01

**Authors:** Eiichi Tokuda, Laura Leykam, Per Zetterström, Thomas Brännström, Peter M Andersen, Stefan L Marklund

**Affiliations:** Department of Medical Biosciences, Umeå University, Umeå, SE 901 85, Sweden; Laboratory of Clinical Medicine, School of Pharmacy, Nihon University, 7-7-1, Narashinodai, Funabashi, Chiba 274-8555, Japan; Department of Medical Biosciences, Umeå University, Umeå, SE 901 85, Sweden; Department of Medical Biosciences, Umeå University, Umeå, SE 901 85, Sweden; Department of Medical Biosciences, Umeå University, Umeå, SE 901 85, Sweden; Department of Clinical Science, Neurosciences, Umeå University, Umeå, SE 901 85, Sweden; Department of Medical Biosciences, Umeå University, Umeå, SE 901 85, Sweden

**Keywords:** Superoxide dismutase-1, Aggregate strains, Strain selection, Coexpression, Bladder control impairment

## Abstract

Mutations in superoxide dismutase-1 (SOD1) are a common cause of amyotrophic lateral sclerosis (ALS). Inheritance is as a rule dominant, but in carriers of the most common mutation, D90A, disease can develop in both homozygous and, more rarely, in heterozygous individuals with unexplained differences in clinical presentation. There is mounting evidence that prion-like spread of SOD1 aggregation is the primary cause of the disease. Two different strains of aggregates have been found to arise in human SOD1 (hSOD1) transgenic mouse models of ALS. Strain A is formed by most mutants including hSOD1^G85R^ and hSOD1^WT^, whereas hSOD1^D90A^ transgenic mice form a distinct strain B in addition to A. To explore the effects of aggregate strain propensities when hSOD1 variants are coexpressed, we generated digenic hSOD1^G85R/WT^ and hSOD1^G85R/D90A^ mice. Coexpression of hSOD1^WT^ considerably shortened the lifespan of hSOD1^G85R^ mice to the extent expected from the neurotoxicities of the variants alone. In contrast, coexpression of hSOD1^D90A^ had a minimal effect on survival, far smaller than expected. Moreover, time from onset to the end stage was markedly prolonged in the hSOD1^G85R/D90A^ mice. Aggregation of hSOD1 developed concomitantly with motor neuron disease, and the aggregates contained large amounts of both coexpressed variants in both digenic models. Our findings suggest that hSOD1^WT^ has high a capacity to coaggregate with mutants and enhance neurotoxicity. Such interactions may be restricted by differences in strain propensities, which may contribute to the primarily recessive inheritance associated with the hSOD1^D90A^ mutation.

## Introduction

Amyotrophic lateral sclerosis (ALS) is characterized by adult-onset degeneration of upper and lower motor neurons. The disease begins focally and then spreads contiguously, resulting in progressive paralysis and death from respiratory failure [1]. Mutations in the gene encoding the ubiquitously expressed free radical scavenging enzyme superoxide dismutase-1 (SOD1) can cause ALS [2] and are found in 1%–9% of patients [3]. Since 1993, over 200 coding mutations in the SOD1 gene have been associated with ALS as a Mendelian dominant trait (http://alsod.iop.kcl.ac.uk/) [4]. A surprising finding is that disease caused by the most prevalent SOD1 mutation, D90A, is most frequently inherited as a recessive trait with a uniform slowly ascending phenotype [5], but may rarely be inherited as a dominant trait with a variable phenotype for the site of first symptom and survival time [6–8].

The SOD1 mutants cause ALS via a gain of neurotoxic function, the character of which is still not fully understood. Cytosolic inclusions containing aggregated SOD1 are hallmarks of ALS both in patients and in transgenic (Tg) animal models expressing mutant human SOD1s (hSOD1) [9]. Using a binary epitope mapping method, we found that two different strains of hSOD1 aggregates, A and B, can arise in transgenic mice that express hSOD1 variants [10, 11]. The hSOD1 variants were found to have different propensities for forming aggregate strains. Strain A aggregates appeared in mice that hemizygously express G93A mutant hSOD1 (hSOD1^G93A^), hSOD1^G85R^ and homozygously express wildtype hSOD1 (hSOD1^WT/WT^). In contrast homozygous hSOD1^D90A/D90A^ mice form a distinct strain B in addition to A. Inoculation of strain A or B aggregate preparations into the spinal cords of adult mice expressing a hSOD1 transgene induced templated hSOD1 aggregations spreading concomitantly with premature fatal motor neuron disease [12]. Seeding effects of spinal cord homogenates from end-stage Tg mice have also been demonstrated in Tg mice as well as in cultured cells and *in vitro* [13–21]. Moreover, a preparation from spinal cord of an ALS patients with a SOD1 mutation has also been found to seed Tg mice [22]. These findings suggest that prion-like spreading of hSOD1 aggregation could be the core pathogenic mechanism of hSOD1-induced ALS.

D90A is the most common ALS-causing hSOD1 mutation and the reason why disease primarily develops in homozygous individuals is still a conundrum [5, 6]. A possibility could be that hSOD1^WT^ can contribute to ALS pathology by readily coaggregating with many mutant hSOD1s but not with hSOD1^D90A^. To explore the potential role of hSOD1^WT^ in ALS, several studies have been carried out in which it has been coexpressed with mutant hSOD1s in Tg mice. The results have been variable: augmented disease was found in most cases (A4V [23], PrP-G37R [24], G85R [25], G93A [23, 26, 27], V103Z [28], T116X [29], L126Z [23]) but negligible effects have also been reported [24, 30]. The absence of effect could, however, be related to the relatively low expression of the hSOD1^WT^ strain used. Human SOD1^WT^ on its own has a neurotoxic potential: mice bred to be homozygous for a transgene insertion (hSOD1^WT/WT^) developed abundant strain A aggregates and a fatal ALS-like disease [10].

Disulfide-reduced hSOD1 monomers lacking the prosthetic metals are poorly stable and seem to be the primary substrates for the nucleation and growth of aggregates *in vitro* and *in vivo* [31–35]. Moreover, mutations in such apo-hSOD1 can significantly influence the population into various transient conformers [36, 37], both with native and non-native structures. Such differences might explain the propensities to form different aggregate strains, as well as the different outcomes of coexpression of hSOD1 variants [38–40]. To investigate this hypothesis, we crossed mice expressing the strain A-propensity hSOD1^G85R^ with mice expressing the strain A-propensity hSOD1^WT^ or with hSOD1^D90A^ which can form both strains B and A. The former combination caused considerable lifespan shortening whereas the effect of the latter combination was far smaller than expected from the neurotoxicities of the mutants alone.

## Results

### Expression of hSOD1^WT^ in hSOD1^G85R^ Tg mice is much more noxious than expression of hSOD1^D90A^

**Figure 1:**
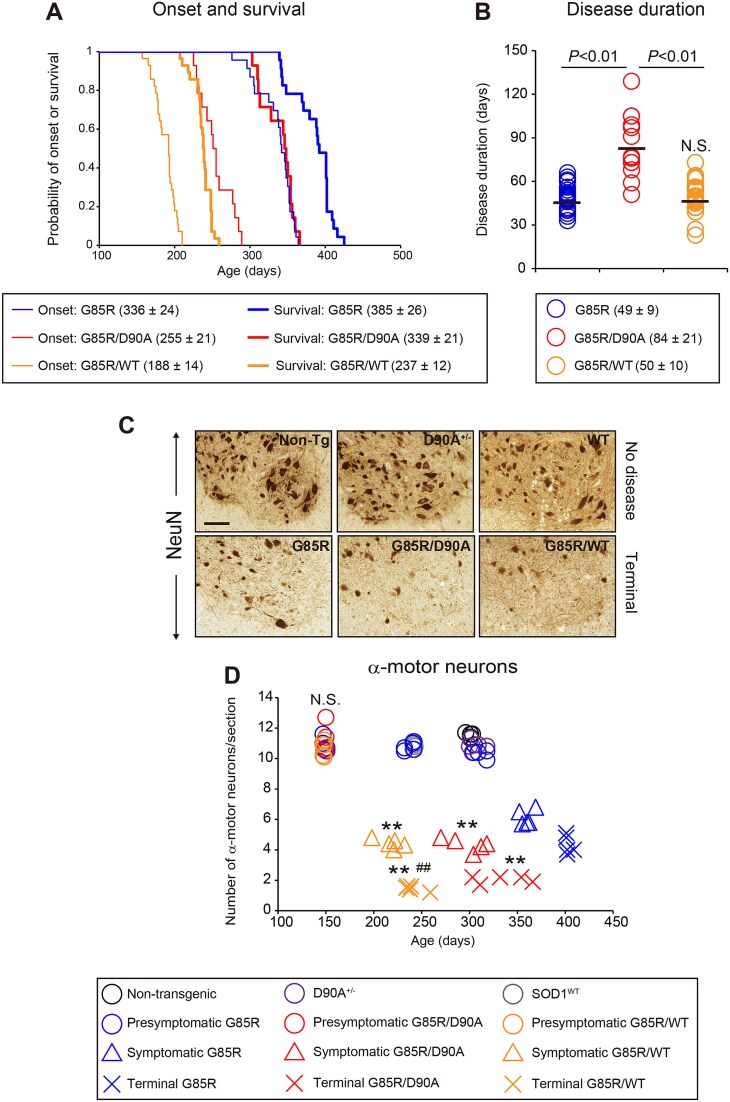
Coexpression of hSOD1s with different aggregate strain propensities accelerates motor neuron death and shortens survival. The disease courses of hSOD1^G85R^ (n = 23; male:Female 12:11), hSOD1^G85R/D90A^ (n = 14; male:Female 10:4), and hSOD1^G85R/WT^ (n = 28; male:Female 13:15) Tg mice were evaluated based on alterations in body weight. (A) Kaplan–Meier curves for disease onset (thin lines) and survival (bold lines). (B) Plots for disease duration, an interval between age at disease onset and age at death. Bars indicate mean values. (C) Representative images of immunohistochemistry for NeuN, a neuronal marker, in lumbar spinal cord sections from mice at terminal stages of the disease. Non-transgenic C57BL/6 (non-Tg) and hSOD1^WT^ mice were used at 240 days, while hemizygous hSOD1^D90A^ (D90A^+/−^) mice were examined at 340 days. Scale bars: 50 μm. (D) Quantification of surviving α-motor neurons in the spinal cords (n = 4–5 per genotype per disease stage). N.S. = not significant (vs. 150-day-old hSOD1^G85R^ mice). ^**^P < 0.01 (vs. the disease stage matched-G85R). ^##^*P* < 0.01 (vs. G85R/D90A at terminal stage). Detailed information on results in Fig. 1D can be found in supplementary table S1.

Mice that hemizygously express the hSOD1^WT^ or hSOD1^D90A^ transgenes do not develop overt motor neuron disease but do so in homozygotes [10, 41, 42]. Here hemizygous hSOD1^G85R^ Tg mice were bred with hSOD1^WT^ and hSOD1^D90A^ Tg mice to generate digenic hSOD1^G85R/WT^ and hSOD1^G85R/D90A^ Tg mice. In both cases, the lifespans of the combined Tg mice were shortened, but the effect of coexpression of hSOD1^WT^ was far greater than that of hSOD1^D90A^ (Fig. 1A). There were also earlier disease onsets (Fig. 1A), but the disease duration was prolonged in the hSOD1^G85R/D90A^ Tg mice (Fig. 1B). Motor neurons were lost earlier in the combined hemizygous Tg mice, and in the end stage, the losses were greater than in the single hemizygous hSOD1^G85R^ Tg mice. (Fig. 1C and D, Supplementary Table S1). The combined Tg mice also showed exacerbation of astrogliosis and microgliosis over the disease course (Supplementary Fig. S1).

The lifespans of the mice in Tg models are highly dependent on hSOD1 expression rates. In some cases, paralytic disease only develops within the short lifespan of mice if the strains are bred to homozygosity for the transgene insertions [10, 42]. For a couple of Tg strains, survival data have been reported for both hemizygous and homozygous mice, including G127X lines 716 and 832 [41], and G93A^del^ (low copy) [26] (Table 1). The lifespans of the homozygotes are approximately halved compared with those of the hemizygotes. This suggests that the aggressiveness of the transgene insertions might tentatively be expressed as additive neurotoxicity indices: 1/lifespan (days). By using such indices, we sought to examine whether the combinations of hSOD1 variants in the double Tg mice combine to cause the neurotoxicity expected from increasing the expression of a single hSOD1 variant (Table 1). The observed lifespan of hSOD1^G85R/WT^ mice was even shorter, although not far from that predicted from the calculations, suggesting that the two strain A-forming variants hSOD1^G85R^ and hSOD1^WT^ combine freely and equally to cause neurotoxicity. In contrast, the lifespan of the hSOD1^G85R/D90A^ Tg mice was far longer than that predicted from the combined indices.

**Table 1:** Predictions of the lifespans of combined hemizygous Tg mice using neurotoxicity indices.

Tg mice	Lifespan (days)		Neurotoxicity index		
	Observed	Predicted	Homozygous	Hemizygous	Combined
D90A homo [42]	407		0.00246	0.00123^*^	
hSOD1^WT^ homo [10]	367		0.00272	0.00136^*^	
G85R hemi (this study)	385			0.00260	
G85R/hSOD1^WT^ (this study)	237	252^*^			0.00396^*^
G85R/D90A (this study)	339	261^*^			0.00383^*^
					
G127X line 716 hemi [41]	477			0.00210	
G127X line 716 homo [41]	250	238^*^	0.00400		0.00420^*^
G127X line 832 hemi [41]	213			0.00469	
G127X line 832 homo [41]	126	106^*^	0.00794		0.00938^*^
Low-copy G93A^del^ hemi [26]	236			0.00428	
Low-copy G93A^del^ homo [26]	146	118^*^	0.00685		0.00847^*^

Tg mice homozygous for transgene insertions live close to half as long as hemizygous Tg mice do, suggesting additive effects. Toxicity indices (1/lifespan) are close to double as high in the homozygotes, suggesting they could be used additively to predict lifespans. We here used toxicity indices for hemizygous hSOD1^G85R^ mice and halved indices for homozygous hSOD1^WT^ and hSOD1^D90A^ Tg mice to predict the lifespans of combined Tg mice.

^*^Calculated data. Hemi = hemizygotes; homo = homozygotes.

### Earlier and enhanced hSOD1 aggregation in the digenic Tg mice

**Figure 2:**
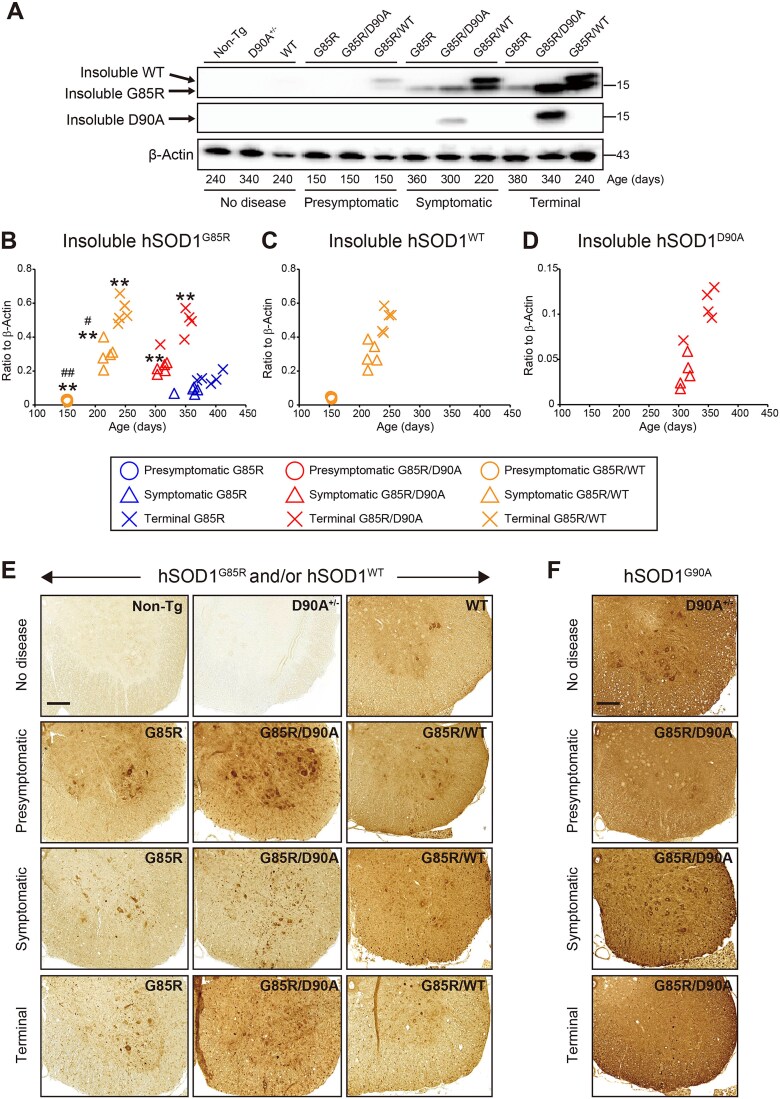
Formation of aggregates in digenic and single Tg mice. Digenic mice were analyzed at three distinct stages of the disease: Presymptomatic (150 days), symptomatic (10% weight loss), and terminal (n = 3–5 per genotype per disease stage). C57BL/6 (non-Tg) and hSOD1^WT^ mice were used at 240 days, while hemizygous hSOD1^D90A^ (D90A^+/−^) mice were examined at 340 days. (A) Western blots for hSOD1^G85R^, hSOD1^WT^, or hSOD1^D90A^ protein in detergent-insoluble fractions from the lumbar spinal cord. β-actin in whole homogenates was used as a loading control. (B-D) scatter plots showing relative levels of insoluble (B) hSOD1^G85R^, (C) hSOD1^WT^, or (D) hSOD1^D90A^ species. ^**^P < 0.01 (vs. the disease stage-matched G85R). ^#^*P* < 0.05, ^##^*P* < 0.01 (vs. the disease stage-matched G85R/D90A). Detailed information on results in Fig. 2B and C can be found in supplementary tables S2 and S3, respectively. (E, F) immunohistochemistry for (E) hSOD1^G85R^ and/or hSOD1^WT^ or (F) hSOD1^D90A^ proteins in lumbar spinal cords from various mouse genotypes (n = 4–5 per genotype per disease stage). In (E) the aa 86–94 hSOD1^WT^ antibody was used, and in (F) the aa 86–94 hSOD1^D90A^ antibody. Scale bars: 100 μm.

To explore whether the shortened survivals of combined transgenes were associated with earlier arising aggregation of the hSOD1 variants, we developed mutually exclusive antibodies that distinguish hSOD1^D90A^ from other hSOD1s in the digenic Tg mice. Rabbits were immunized with the aa 86–94 peptides in the hSOD1^D90A^ and hSOD1^WT^ sequences, respectively. The resulting hSOD1^D90A^-antibody recognized hSOD1^D90A^ protein but not other hSOD1s or endogenous murine SOD1, whereas the hSOD1^WT^ antibody did not react with the hSOD1^D90A^ protein (Supplementary Fig. S2). Using these antibodies, we analyzed the insoluble fraction from spinal cords of digenic Tg mice at different disease stages. We found that the shortened survivals of the mixed hemizygous hSOD1^G85R/WT^ and hSOD1^G85R/D90A^ Tg mice were associated with both earlier arising hSOD1^G85R^ aggregation and higher levels in the spinal cords in the end stage (Fig. 2A and B, Supplementary Table S2). There were also parallel increases in aggregations of hSOD1^WT^ and hSOD1^D90A^ (Fig. 2A, C, D, and Supplementary Table S3). The amounts of aggregated hSOD1^G85R^ and hSOD1^WT^ in the hSOD1^G85R/WT^ Tg mice were nearly equal from the presymptomatic stage (150 d) through the symptomatic and terminal stages (Fig. 2A to C, Supplementary Tables S2 and S3). Because of the specific antibody used for determination of aggregated hSOD1^D90A^, we cannot directly compare with amounts of hSOD1^G85R^ in the hSOD1^G85R/D90A^ Tg mice. Increased amounts of aggregated hSOD1s were also apparent in studies of spinal cord sections by immunohistochemistry (Fig. 2E and F).

### Strain a aggregates are formed in hSOD1^G85R/WT^ and hSOD1^G85R/D90A^ Tg mice

**Figure 3:**
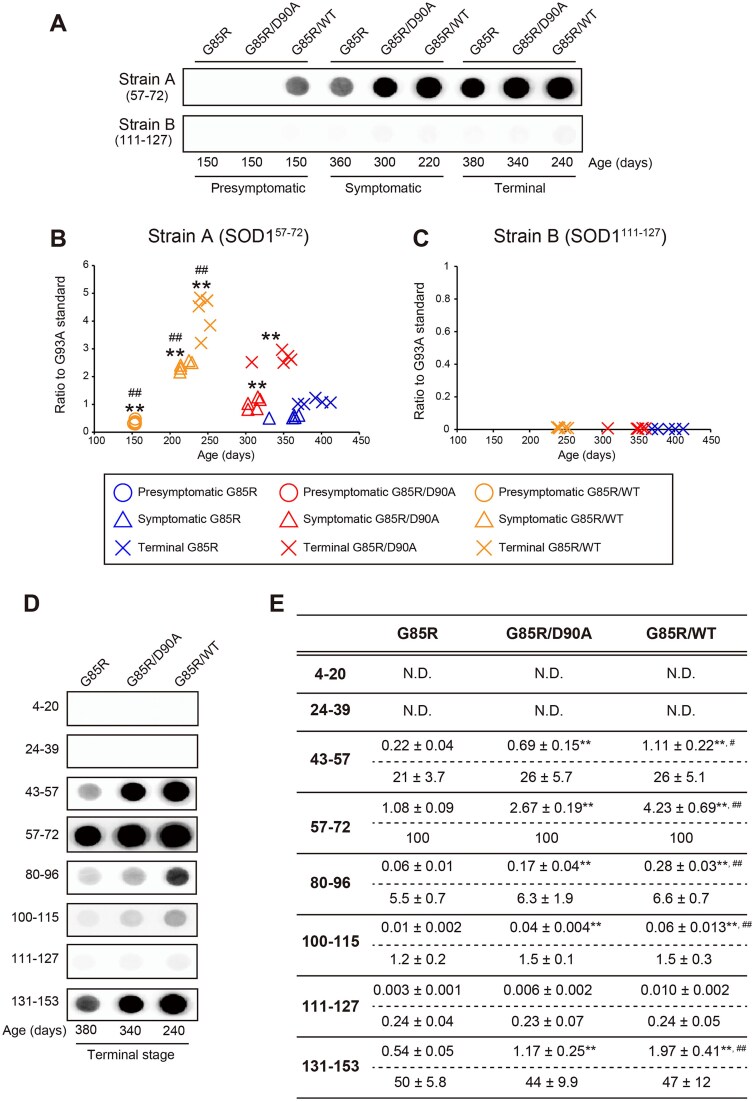
Coexpression of hSOD1s with distinct aggregate strain propensities induces strain a, but not strain B. (A) Filter-trapped assays for hSOD1 strain a or strain B aggregates in the lumbar spinal cords of the digenic mice at three distinct stages of the disease (n = 3–5 per genotype per disease stage). (B, C) quantification of the amounts of (B) strain a and (C) strain B aggregates. ^*^^*^P < 0.01 (vs. the disease stage matched-G85R). ^##^*P* < 0.01 (vs. the disease stage-matched G85R/D90A). Detailed information on results in Fig. 3B can be found in supplementary table S4. (D) Representative examples of epitope-mapping assays for conformational patterns of hSOD1 aggregates in terminally ill mice. (E) Results of epitope mapping of amounts of hSOD1 aggregates in terminally ill mice using the hSOD1^G93A^ standard, and patterns expressing the reactivities with the antibodies compared with the aa 57–72 antibody set to 100%. ^*^^*^P < 0.01 (vs. G85R). ^#^*P* < 0.05, ^##^*P* < 0.01 (vs. G85R/D90A). N.D. = not detected.

In binary epitope mapping, multiple antibodies raised against peptides covering the hSOD1 sequence are used to stain filter-captured aggregates [11]. The antibodies used only react with disordered hSOD1. They do not stain native SOD1 or the ordered core of hSOD1 aggregates/fibrils or segments otherwise hidden but react avidly with non-recruited disordered solvent-exposed sequence elements. To quantify the aggregation, we used the aa 57–72 antibody which primarily reacts with strain A aggregates (Fig. 3A and B) [11]. The increases in aggregation followed the same time courses as when analyzed as detergent-resistant aggregates (Fig. 2B to D, and Supplementary Table S4). In strain B aggregates, the entire mid and C-terminal portions of hSOD1 protrude into the solvent in a disordered manner resulting in strong reaction with antibodies targeting these epitopes. However, there was no evidence for the formation of B aggregates as determined using the aa 111–127 antibody (Fig. 3C). To more closely examine aggregate strain patterns, spinal cords from terminal single and double Tg mice were examined with eight anti-peptide antibodies covering the hSOD1 sequence (Fig. 3D and E). The amounts of aggregates were greater in the combined hemizygous mice than in the single Tg hSOD1^G85R^ mice, but the normalized antibody profiles showed typical strain A patterns and were virtually identical in the three strains.

To further explore the structure of the aggregates, we used the aa 86–94 hSOD1^WT^ and hSOD1^D90A^-specific antibodies for epitope mapping (Supplementary Table S5). There were small but significant reactions. The aa 86–94 (D90A) antibody reacted only with the hSOD1^G85R/D90A^ samples, whereas the aa 86–94 (WT) antibody reacted with the hSOD1^G85R^, hSOD1^G85R/D90A^, hSOD1^G85R/WT^ and hSOD1^WT/WT^ samples. Together with the reactions with the aa 80–96 antibody (Fig. 3D and E), the results suggest that the sequence element that contains the hSOD1^G85R^ and hSOD1^D90A^ mutations does not form a part of an ordered cross β-sheet structure in the core of the aggregate fibrils.

### The exacerbated disease is not associated with increased levels of the soluble fraction of SOD1 variants

In the presymptomatic stage, at 150 d, there were no differences in total or soluble hSOD1^G85R^ protein between single Tg hSOD1^G85R^ mice and the hSOD1^G85R/D90A^ mice. In the symptomatic and terminal stages, the total hSOD1^G85R^ protein in the hSOD1^G85R/D90A^ Tg mice rose because of the abundant aggregates formed, whereas no significant change could be seen in the hSOD1^G85R^ mice. The soluble hSOD1^G85R^ protein decreased moderately in the later stages in the hSOD1^G85R^ mice but not in the digenic hSOD1^G85R/D90A^ mice (Supplementary Fig. S3A, B, and C). The hSOD1^D90A^ protein is 11-fold more abundant than the hSOD1^G85R^ protein in hemizygous Tg mice [43]. In the hSOD1^G85R/D90A^ Tg mice no changes in total and soluble amounts of the hSOD1^D90A^ protein were discernible over the disease course, nor were they different from the levels in single Tg hSOD1^D90A^ mice (Supplementary Fig. S3D, E, and F).

We previously found that the hSOD1^WT^ level is about 27 times as high as the hSOD1^G85R^ protein in single Tg mice [43], and it was therefore not possible to distinguish the two in hSOD1^G85R/WT^ Tg mice using their different sodium dodecyl sulfate–polyacrylamide gel electrophoresis mobilities (Supplementary Fig. S4) [44, 45]. There were, however, no significant changes in total and soluble hSOD1 over the disease course in the hSOD1^G85R/WT^ mice (Supplementary Fig. S4). Thus, there is no evidence that the coexpressions overload the capacity to degrade the hSOD1^G85R^ protein or the other variants, explaining the exacerbated motor neuron disease.

Total, soluble, and aggregated endogenous murine SOD1 were also measured over the disease course in the various single and combined hemizygous hSOD1 Tg models (Supplementary Fig. S5). As previously reported [43], there are lower levels in mice expressing hSOD1^D90A^. This is apparently an effect of the insertion site of the transgene array in the genome: no reduction was observed in another hSOD1^D90A^ Tg line with comparable hSOD1^D90A^ expression [43]. Apart from this deviation, there were no changes in total or soluble murine SOD1, nor were any aggregates of the protein found. The results suggest that there are no effects on the synthesis of murine or human SOD1 variants *per se* during the disease courses in the various Tg models.

### Urinary bladder dysfunction is not specific for hSOD1^D90A^ or strain B aggregates

**Figure 4:**
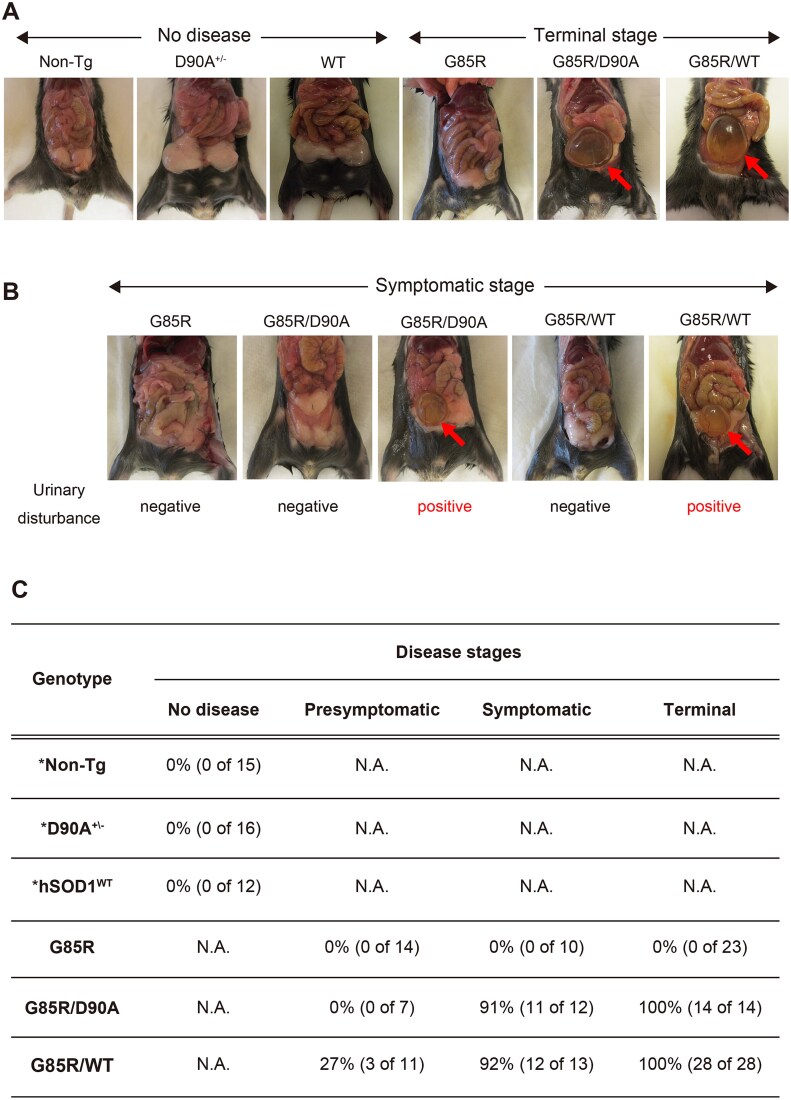
Occurrence of urinary bladder disturbances in digenic mice. Representative images of bladder disturbances (arrows) in (A) terminally ill and (B) symptomatic double and single Tg mice. (C) Number of mice that developed bladder disturbance. ^*^ = C57BL/6 J (non-Tg) and hSOD1^WT^ mice were analyzed at 240 days of age, whereas hemizygous hSOD1^D90A^ (+/−) mice were examined at 300 days. N.A. = not applicable.

Urgence of micturition because of impaired control of the detrusor muscle is uncommon among ALS patients in general, but frequent among ALS patients homozygous for the hSOD1^D90A^ mutation [5]. Such dysfunction is also regularly observed in end-stage hSOD1^D90A/D90A^ Tg mice [42]. It has not been reported to occur in hSOD1^G85R^ [46], hSOD1^WT/WT^ mice [10, 26] or other mutant hSOD1 Tg models. Against this background, the appearance of the urinary bladder was recorded in the present Tg mouse combinations (Fig. 4).

The hSOD1^G85R^ Tg mice showed no bladder disturbances at any of the disease stages (Fig. 4C). In contrast, all the end stage hSOD1^G85R/D90A^ and hSOD1^G85R/WT^ Tg mice displayed distended urinary bladders (Fig. 4A and C). This was also observed in most of the symptomatic mice, and in some of the hSOD1^G85R/WT^ Tg mice already before onset of paretic symptoms (Fig. 4B and C). We conclude that, at least in the mouse Tg models, there is no specific relationship between bladder dysfunction and the hSOD1^D90A^ mutation or with strain B aggregation.

## Discussion

Here we show that coexpression of hSOD1^WT^ or hSOD1^D90A^ with hSOD1^G85R^ in Tg mice results in earlier onsets and shortened survivals compared with mice expressing hSOD1^G85R^ alone. Although not shown here directly, we suggest that the mechanism involves direct combination of hSOD1^WT^ or hSOD1^D90A^ with hSOD1^G85R^ to accelerate the nucleation and growth of aggregate fibrils. In support of this notion, Tg mice that express hSOD1^WT^ together with murine SOD1^G86R^ show the same ALS-like phenotype as do mice expressing the murine mutant alone [47]. There is no evidence that hSOD1 can coaggregate with murine SOD1 (Supplementary Fig. S5) [11, 48, 49].

Mutations can significantly influence transiently appearing conformations of poorly stable apo hSOD1 monomers [36, 37]. Such differences have been found to influence the propensities of the variants to form different aggregate conformers *in vitro* as well as in cultured cells [38–40]. A primary goal of the present study was to determine whether such diversities in strain propensities would influence the outcomes of coexpression of mutants in Tg mice. This seems to be the case: the two strain A forming variants hSOD1^G85R^ and hSOD1^WT^ showed additive neurotoxicities (Fig. 1 and Table 1). In contrast, coexpression of hSOD1^G85R^ with hSOD1^D90A^, which alone forms both strains B and A, caused much less shortening of the lifespan. In both cases the aggravated motor neuron disease was associated with earlier arising hSOD1 aggregation, and higher levels in the end stage (Figs 2 and 3). Moreover, in the hSOD1^G85R/WT^ Tg mice the amounts of the variants in the aggregates were nearly equal. Owing to the use of different antibodies for quantifications, we could not directly compare amounts of the variants in the hSOD1^G85R/D90A^ Tg mice, but large quantities of both mutants appeared in aggregates in symptomatic and end-stage mice. There were no changes in the amounts of soluble hSOD1 variants before disease onset (Supplementary Figs S3 and S4), nor was there any effect on the endogenous murine SOD1 at any stage (Supplementary Fig. S5). This indicates that the coexpressions did not per se influence the turnover of SOD1 in the spinal cords.

The expression rates/mRNA and hSOD1 protein contents are both about 2.5 times higher in hemizygous hSOD1^WT^ mice than in hemizygous hSOD1^D90A^ mice [42, 43]. In some previous studies with coexpression of hSOD1^WT^ with mutant hSOD1s, the rate of expression of the hSOD1^WT^ transgene has been important for the exacerbations [24, 25, 28, 30]. This bulk effect of hSOD1 expression may have influenced the differences between the current hSOD1^WT^ and hSOD1^D90A^ coexpressions.

In the binary epitope mapping, both the aa 80–96 and the two variant-specific aa 86–94 antibodies were found to react weakly but significantly with the aggregates. This indicates that the sequence element harboring the mutations is not part of an ordered cross β-sheet, hence mismatches between the wildtype and mutant amino acids in such a structure would not explain the outcomes of the various crosses. The weak reactions with the bulky antibodies, however, indicates steric hindrance and proximity to a fibril core. We propose that differences in conformations of disulfide-reduced apo hSOD1 monomers could influence the propensities to coaggregate by affecting encounter complexes, nucleation or recruitment to growing fibrils. The aggregates formed in hSOD1^G85R/WT^ Tg mice showed typical strain A patterns as analyzed by binary epitope mapping (Fig. 3). So did the aggregates in hSOD1^G85R/D90A^ mice, and the aggregate patterns in the coexpression mice appeared identical to those in hSOD1^G85R^ Tg mice. Strain A aggregates can form in hSOD1^D90A/D90A^ Tg mice, and for some reason strain A aggregation prevailed in the hSOD1^G85R/D90A^ mice. This is not because the hSOD^G85R^ protein cannot form strain B aggregates (Fig. 3A and B): inoculation of such aggregates into the spinal cords of hSOD1^G85R^ Tg mice initiates templated strain B aggregation [12]. The growth rate is, however, 50% slower than hSOD^G85R^ aggregation initiated by inoculation of strain A aggregates [12]. The growth of strain A aggregate fibrils by addition of disordered hSOD1^G85R^ and hSOD1^D90A^ monomers may have been somewhat impeded, and relatively more affected than the nucleation phase, explaining the slower disease progression in the hSOD^G85R/D90A^ mice (Fig. 1B).

The amounts of hSOD1 aggregates in end-stage spinal cords were larger in the combined Tg mice than in hSOD1^G85R^ Tg mice (Figs 2 and 3) and the losses of motor neurons were greater (Fig. 1C and D). The explanation is not obvious. Paralysis in hSOD1 Tg mice can be caused by both loss of motor neurons and dysfunction [50]. The latter mechanism may play a proportionally greater role in the hSOD1^G85R^ Tg mice. These mice were also older when they approached the terminal stage and might be more vulnerable to accumulation of hSOD1 aggregates and loss of motor neurons. Even though the current results suggest that hSOD1 aggregation is the primary cause of disease, the identities of the principal harmful species are still unknown. There is likely a continuum of aggregate species from small fibrils to larger aggregates. *In vitro* cell culture studies suggest that small fibrils could be more toxic [51]. Furthermore, aggregates gradually become sequestered into inclusion bodies and likely rendered less noxious [52]. The fragilities and interaction properties of aggregate fibrils composed of a single or two hSOD1 variants may differ, leading to different size distributions and somewhat different cellular effects.

The current findings suggest that hSOD1^WT^ has the potential to contribute to the disease in ALS patients who are heterozygous for hSOD1 mutations. A potentiation is shown here in hSOD1^G85R^ Tg mice, and similar effects have previously been reported for ALS-linked hSOD1 mutants, including G93A [23, 26, 27], G37R [24, 28], L126Z [23, 24], A4V [23], and G85R [25] as well as for experimental SOD1 mutants such as V103Z [28] and T116X [29]. Our results further suggest that such potentiation might be restricted by the aggregation strain propensities. *In vitro* and when overexpressed in cultured cells aggregation of hSOD1 shows a great plasticity and many different structures are formed that vary between mutants [11, 21, 38, 39, 53, 54]. The conditions in the CNS, however, constrains the aggregation process and among the limited number of hSOD1 variants so far analyzed we have only found the A and B strain patterns to be formed [11]. Other strain propensities should exist among the additional 200-plus ALS-associated hSOD1 mutants, and these may to variable extents influence the putative interactions with hSOD1^WT^ in the CNS.

In conclusion, the previous and our present findings suggest that hSOD1^WT^ has high capacity to coaggregate with mutants and enhance neurotoxicity. Such interactions may be restricted by different strain propensities, which could be one of the reasons why ALS primarily develops in individuals homozygous for the hSOD1^D90A^ mutation.

## Materials and methods

### Generation and monitoring of double transgenic mice expressing various human SOD1s

The animal protocols were approved by the Umeå Ethical Committee on Animal Experiments. We used three different lines of transgenic mice carrying human SOD1: WT (strain name: B6SJL-Tg(SOD1)2Gur/J; stock number: 002297) [55], G85R [46], and D90A [42]. The human *SOD1* transgene, contained within a 12 kb genomic DNA fragment that was excised by *Eco*RI and *Bam*HI, in all Tg lines is expressed under the control of its endogenous human SOD1 promoter, leading to ubiquitous expression of the hSOD1 protein. They were backcrossed into a C57BL/6 J background for more than 30 generations; thus, they are congenic lines. For the generation of double transgenic mice, male hemizygous hSOD1^G85R^ mice were crossed with either female hemizygous hSOD1^D90A^ or hSOD1^WT^ mice. To identify the genotype of the offspring, the hSOD1 transgene was detected using PCR with the following primers: 5’-CAT CAG CCC TAA TCC ATC TGA-3′ for forward, and 5’-CGC GAC TAA CAA TCA AAG TGA-3′ for reverse (236-bp). The SOD1^G85R^ and SOD1^D90A^ mutations were directly identified using a sequence analysis as described previously [56]. The genotype was also validated by using Western blots with specific antibodies that distinguish hSOD1^G85R^ from hSOD1^D90A^ (See “*Generation of hSOD1^WT^ and hSOD1^D90A^- specific antibodies*”).

The disease course of the mice was evaluated according to criteria based on changes in body weight [57]. Disease onset was regarded as the time when each mouse reached its peak weight. The endpoint was defined as the age at which a mouse was unable to right itself within 5 s after being pushed onto its side [58]. The duration of disease was regarded as the period from disease onset until the endpoint. In some of the mice the paralysis was more prominent in the forelimbs. In these cases, the end stage was set to when paralysis of the forelimbs was so extensive that the mice could not eat wet food or developed an eye infection.

### Generation of hSOD1^WT^ and hSOD1^D90A^- specific antibodies

To distinguish the hSOD1^D90A^ protein from other hSOD1 variants (especially hSOD1^WT^ and hSOD1^G85R^), we developed mutually exclusive anti-hSOD1 antibodies. The synthetic peptides NVTADKDGV (amino acids (aa) 86–94 in hSOD1^WT^) NVTAAKDGV (aa 86–94 hSOD1^D90A^, the mutational residue underlined in bold) coupled to keyhole limpet hemocyanin were used to immunize rabbits. The antisera from the rabbits immunized with each peptide were purified using Protein A-Sepharose (Cytiva, Marlborough, MA, USA), followed by antigen affinity purification on SulfoLink Resin conjugated with either of the immunization peptides (Pierce, Waltham, MA, USA).

### Collection and fractionation of murine tissues

The hSOD1^G85R/D90A^, hSOD1^G85R/WT^, and their single transgenic littermates were analyzed at three distinct stages of the disease: a presymptomatic stage (150 days), a symptomatic stage (10% weight loss), and a terminal stage (n = 3–5 per genotype per disease stage). For the collection of tissues, mice were deeply anesthetized with intraperitoneal injections of sodium pentobarbital. The lumbar spinal cord was immediately dissected, frozen in liquid nitrogen, and stored at −80°C until use.

The spinal cords of mice were extracted as described previously [59]. Briefly, the lumbar spinal cords were homogenized in 25 volumes of ice-cold phosphate buffered saline (PBS, pH 7.0) containing 1% (v/v) Nonidet P-40 and EDTA-free Complete® protease inhibitor cocktail (Roche Applied Science, Penzberg, Germany). A portion of the homogenates was centrifuged at 20000 × *g* for 30 min at 4°C. The supernatants and the pellets were collected as detergent-soluble and -insoluble fractions, respectively. The pellets were washed three times in double the original volume of homogenizing buffer. Protein concentration of the extracts was determined using a Bradford assay according to the manufacturer’s directions (Bio-Rad, Hercules, CA, USA). The supernatants were used for western blots as described below.

### Western blots

Tissue extracts (20 μg protein/lane) were electrophoresed on Criterion® TGX Any kD or 18% gels (Bio-Rad) and blotted onto polyvinylidene difluoride membranes (Cytiva). The membranes were treated with blocking solution containing 0.5% (w/v) ECL Advance Blocking Reagent (Cytiva) in Tris-buffered saline (pH 7.5) with 0.01% (v/v) Tween 20. For SOD1 analysis, we used rabbit anti-hSOD1^D90A^ (0.001 μg/ml), rabbit anti-hSOD1^WT^ (0.01 μg/ml), or rabbit anti-murine SOD1 antibodies (0.1 μg/ml, raised against a peptide corresponding to aa 24–36 in murine SOD1) [43]. β-Actin (1:50000; MAB1501R; Millipore, Burlington, MA, USA) was used as a loading control. As secondary antibody, horseradish peroxidase-conjugated anti-rabbit or anti-mouse IgG (1:25000; Agilent Dako, Santa Clara, CA, USA) was used. The immunoreaction was visualized using an ECL Select reagent (Cytiva). The chemiluminescence of the blots was quantified using Quantity One software (Bio-Rad).

### Binary epitope-mapping assay for quantification and structural analysis of hSOD1 aggregates

A detailed description of the protocol and background to the binary epitope-mapping method is found in our previous paper [11]. The protocol is based on multiple polyclonal rabbit antibodies (Ra-Abs) raised against short peptides covering the 153 aa long hSOD1 subunits. The reactions of the antibodies with aggregates captured on filter is determined. Since the configurational space of short peptides is very large, their randomly induced antibody-eliciting epitopes are unlikely to match defined ordered structures in proteins and in the cores of aggregate fibrils. The antibodies used lack reactivity with natively folded hSOD1. In contrast, all react avidly with denatured/disordered hSOD1, in which the corresponding peptide segments can adapt to the antigen-binding sites. In reactions with fibrils/aggregates, the binding of the anti-peptide antibodies is essentially ‘binary’. There is no response to the ordered core of protein aggregates/fibrils or to segments otherwise hidden. Non-recruited sequence elements, which have lost their native contacts and therefore are disordered, will react with the antibodies.

Aggregates in serial 1 + 1 dilutions of the preparations to be analyzed were trapped in 0.2 μm cellulose acetate filters in a dot-blot apparatus. All samples were run in duplicate. The filters were washed three times with PBS. They were then cut into slices, incubated with the eight antibodies overnight and finally developed similarly to western immunoblots. Chemiluminescence of the blots was recorded in a ChemiDoc Touch Imaging System (BioRad) and analyzed with ImageLab software. To allow comparison and quantification, one homogenate of spinal cord from an end-stage hSOD1^G93A^ Tg mouse, kept frozen in aliquots, is designated as a standard (set to 1) and run in one or two lanes on all the filters and stained with the 57–72 Ra-Ab. All the blots of all the preparations with all the antibodies were quantified against this standard. To facilitate comparison of BEM staining patterns, the staining intensities of the eight antibodies with individual preparations were normalized against the staining of that preparation with the 57–72 Ra-Ab (taken as 100%).

### Immunohistochemistry

Immunohistochemical studies were performed as described previously [58]. Lumbar spinal sections (6 μm thick) from double and single transgenic hSOD1 mice at three different disease stages (n = 4–5 per genotype per disease stage) were immunostained with the following primary antibodies: rabbit anti-hSOD1^WT^ (0.1 μg/mL), rabbit anti-SOD1^D90A^ (0.02 μg/mL), mouse anti-glial fibrillary acidic protein (GFAP) cocktail (0.01 μg/ml; 556 330; BD Biosciences, Franklin Lakes, NJ, USA), and rabbit anti-ionized calcium-binding adapter molecule 1 (Iba1) (0.05 μg/ml; 019–19 741; Fujifilm Wako Pure Chemicals, Osaka, Japan). The immunoreaction was amplified using the VECTASTAIN® ABC Kit (Vector Laboratories, Newark, CA, USA) and was detected using 3,3′-diaminobenzidine (Dako Agilent) as the chromogen. The sections were imaged using a Pannoramic 250 Flash II scanner (3D Histech Ltd, Budapest, Hungary).

Counting of α-motor neurons was performed as described previously [58]. Every tenth lumbar section of the spinal cord (L1–L3) was immunostained with mouse anti-NeuN antibody (1 μg/ml; MAB377; Millipore) to avoid repeated counting of the same α-motor neurons. The size of the soma of NeuN-positive neurons was measured using Pannoramic Viewer software (3D Histech Ltd). The number of α-motor neurons (>400 μm^2^) in the ventral horn was counted in 10 sections per mouse (n = 3–5 per genotype per disease stage).

### Statistics

The data are presented as means ± S.D. All statistical tests were performed with Statcel 3 software (OMS Publishing Inc., Saitama, Japan). Temporal changes in the body weight of the mice were analyzed using repeated-measures ANOVA. The disease onset and survival of mice were compared using Kaplan–Meier analysis with the log-rank test. Multiple group comparisons were performed using one-way ANOVA followed by the Tukey–Kramer *post-hoc* test. Statistical significance was defined as *P* < 0.05. The ‘n’ values indicate the numbers of animal individuals but not replicate measurements on one sample. All biochemical and histopathological studies were repeated at least twice to confirm the results.

## Supplementary Material

Supplementary_Fig_S1_ddaf088

Supplementary_Fig_S2_ddaf088

Supplementary_Fig_S3_ddaf088

Supplementary_Fig_S4_ddaf088

Supplementary_Fig_S5_ddaf088

Supplementary_Table_S1_ddaf088

Supplementary_Table_S2_ddaf088

Supplementary_Table_S3_ddaf088

Supplementary_Table_S4_ddaf088

Supplementary_Table_S5_ddaf088
