# Supplementary material for: Diverse effects of coexpression of human SOD1 variants on motor neuron disease
Source: Hum Mol Genet. 2025 Jun 1;34(16):1380–91. doi: 10.1093/hmg/ddaf088 (PMC12361113; doi:10.1093/hmg/ddaf088)
Supplement: Supplementary_Fig_S1_ddaf088 [file supplementary_fig_s1_ddaf088.docx]

**Supplementary Figure S1**


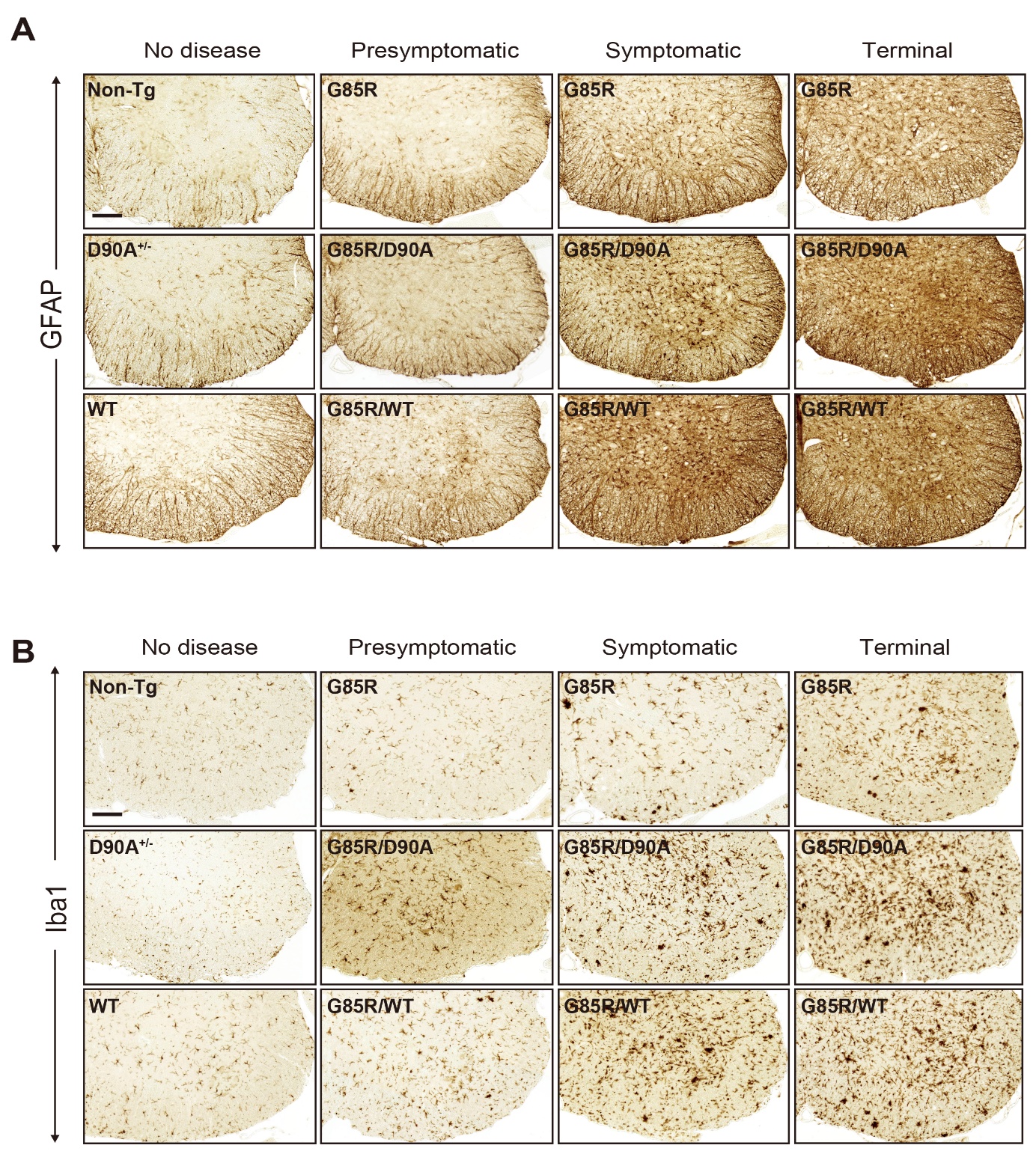


**Supplementary Fig. S1 Coexpression of hSOD1 variants exacerbates the activation of astrocytes and microglia throughout the disease course**

Lumbar spinal cords were dissected from various mouse genotypes at three different stages of the disease: presymptomatic (150 days), symptomatic (10% weight loss), and terminal (n = 4-5 per genotype per disease stage). Immunohistochemistry for (**A**) GFAP and (**B**) Iba1, which are markers of astrocytes and microglia, respectively. Non-transgenic C57BL/6 (non-Tg) and hSOD1^WT^ mice were used at 240 days, whereas hemizygous hSOD1^D90A^ (D90A^+/-^) mice were examined at 340 days. Scale bars: 100 μm.
