# Supplementary material for: Diverse effects of coexpression of human SOD1 variants on motor neuron disease
Source: Hum Mol Genet. 2025 Jun 1;34(16):1380–91. doi: 10.1093/hmg/ddaf088 (PMC12361113; doi:10.1093/hmg/ddaf088)
Supplement: Supplementary_Fig_S2_ddaf088 [file supplementary_fig_s2_ddaf088.docx]

**Supplementary Figure S2**


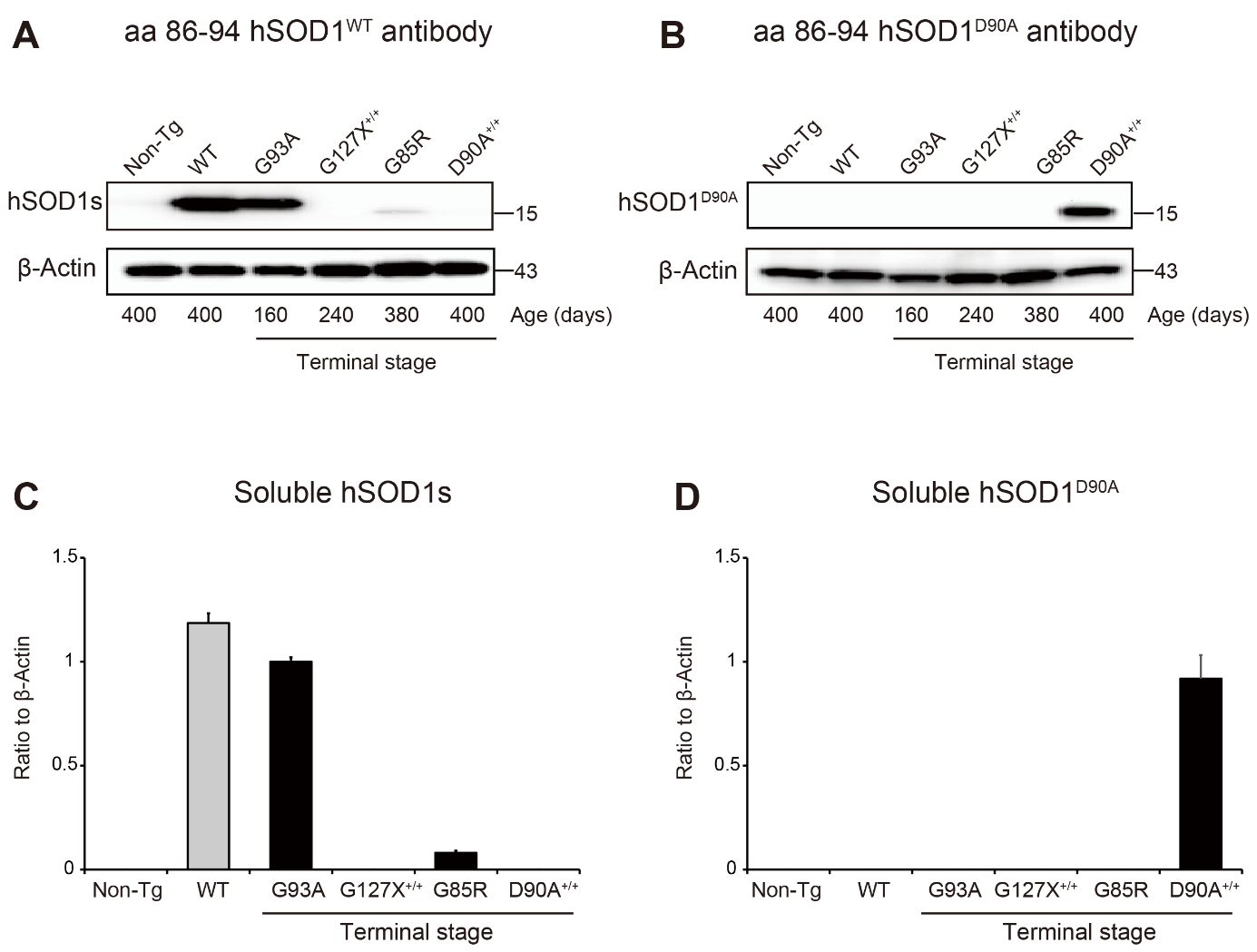


**Supplementary Fig. S2 Characteristics of novel antibodies against hSOD1^WT^ and hSOD1^D90A^**

Lumbar spinal cords were harvested from terminally ill mice carrying mutated hSOD1 (n = 3 per genotype). Non-transgenic C57BL/6 (non-Tg) and hSOD1^WT^ mice were used at 400 days of age. Western blots with the newly developed antibodies against (**A**) hSOD1^WT^ (aa 86-94 hSOD1^WT^) or (**B**) hSOD1^D90A^ (aa 86-94 hSOD1^D90A^) to detect hSOD1s or hSOD1^D90A^ proteins in detergent-soluble fractions from the spinal cords, respectively. β-Actin was used as a loading control. Relative levels of (**C**) anti-hSOD1^WT^ antibody-reactive hSOD1s or (**D**) hSOD1^D90A^ protein.
