# Supplementary material for: Diverse effects of coexpression of human SOD1 variants on motor neuron disease
Source: Hum Mol Genet. 2025 Jun 1;34(16):1380–91. doi: 10.1093/hmg/ddaf088 (PMC12361113; doi:10.1093/hmg/ddaf088)
Supplement: Supplementary_Fig_S3_ddaf088 [file supplementary_fig_s3_ddaf088.docx]

**Supplementary Figure S3**


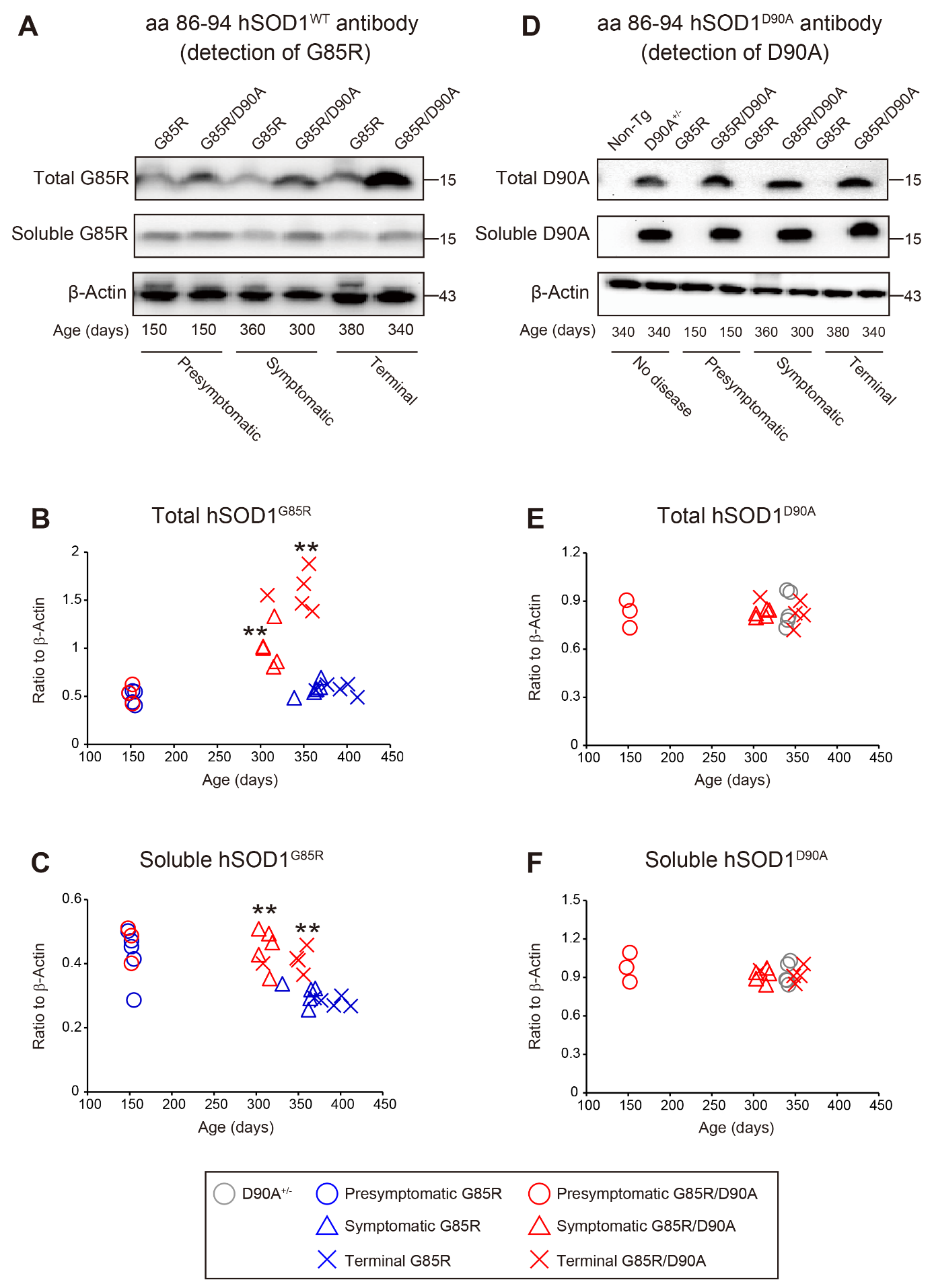


**Supplementary Fig. S3 Amounts of total and soluble hSOD1^G85R^ and hSOD1^D90A^ proteins in digenic mice**

Lumbar spinal cords were dissected from digenic mice and their littermates at three distinct stages of the disease: presymptomatic (150 days), symptomatic (10% weight loss), and terminal (n = 3-5 per genotype per disease stage). Non-transgenic C57BL/6 (non-Tg) and hSOD1^WT^ mice were used at 240 days, while hemizygous hSOD1^D90A^ mice were examined at 340 days. (**A**, **D**) Western blots for (**A**) hSOD1^G85R^ and (**D**) hSOD1^D90A^ in whole homogenate and detergent-soluble fractions from the spinal cords of hSOD1^G85R/D90A^ mice and their littermates. β-Actin in whole homogenates was used as a loading control. Scatter plots showing relative expression levels of (**B**, **E**) total and (**C**, **F**) soluble hSOD1 proteins, which were calculated as the ratio to β-actin. ***P*<0.01 (vs. the disease stage-matched G85R).
