# Supplementary material for: Diverse effects of coexpression of human SOD1 variants on motor neuron disease
Source: Hum Mol Genet. 2025 Jun 1;34(16):1380–91. doi: 10.1093/hmg/ddaf088 (PMC12361113; doi:10.1093/hmg/ddaf088)
Supplement: Supplementary_Fig_S4_ddaf088 [file supplementary_fig_s4_ddaf088.docx]

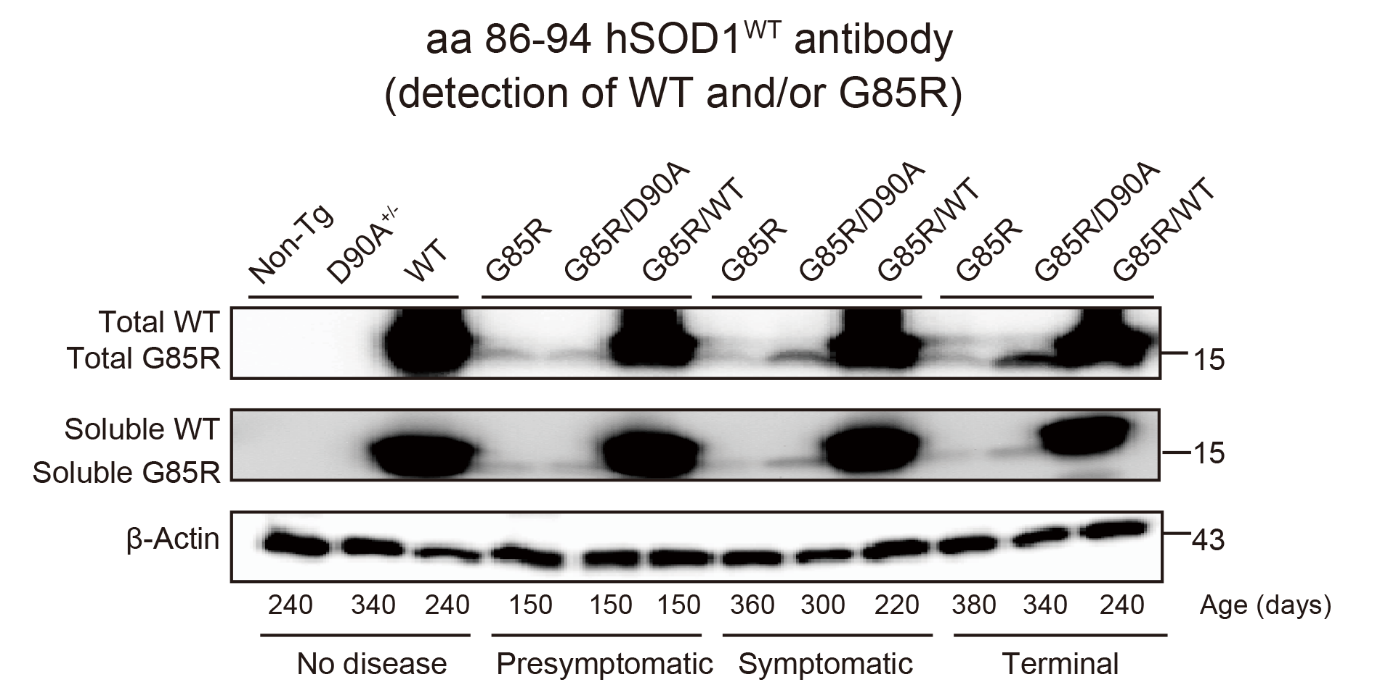
**Supplementary Figure S4**

**Supplementary Fig. S4 Amounts of total and soluble hSOD1^G85R^ and hSOD1^WT^ protein in digenic mice**

The lumbar spinal cords were dissected from digenic mice and their littermates at three distinct stages of the disease: presymptomatic (150 days), symptomatic (10% weight loss), and terminal (n = 3-5 per genotype per disease stage). Non-transgenic C57BL/6 (non-Tg) and hSOD1^WT^ mice were used at 240 days, while hSOD1^D90A^ mice were examined at 340 days. Western blots with the aa 86-94 hSOD1^WT^ antibody for the detection of hSOD1^G85R^ or hSOD1^WT^ in whole homogenate and detergent-soluble fractions from the spinal cords. β-Actin in whole homogenates was used as a loading control.
