# Supplementary material for: Diverse effects of coexpression of human SOD1 variants on motor neuron disease
Source: Hum Mol Genet. 2025 Jun 1;34(16):1380–91. doi: 10.1093/hmg/ddaf088 (PMC12361113; doi:10.1093/hmg/ddaf088)
Supplement: Supplementary_Fig_S5_ddaf088 [file supplementary_fig_s5_ddaf088.docx]

**Supplementary Figure S5**


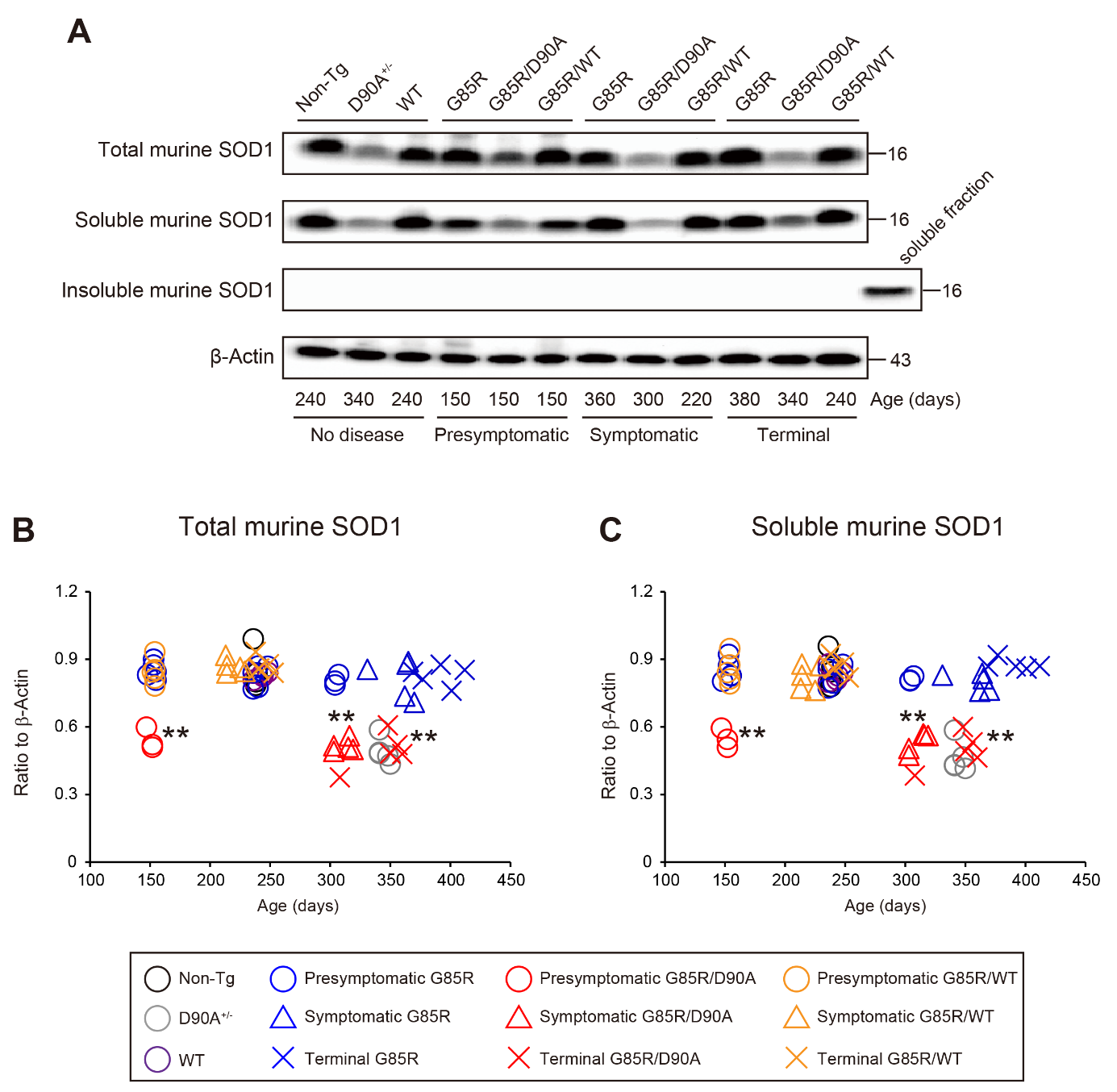


**Supplementary Fig. S5 Expression and aggregation of hSOD1 variants does not influence the endogenous murine SOD1**

The lumbar spinal cords were dissected from digenic mice and their littermates at three distinct stages of the disease: presymptomatic (150 days), symptomatic (10% weight loss), and terminal (n = 3-5 per genotype per disease stage). Non-transgenic C57BL/6 (non-Tg) and hSOD1^WT^ mice were used at 240 days, while hemizygous hSOD1^D90A^ mice were examined at 340 days. A murine-specific aa 24-36 antibody was used for the analyses. (**A**) Western blots for endogenous murine SOD1 protein in whole homogenate, detergent-soluble, and detergent-insoluble fractions from the spinal cords. For analysis of murine SOD1 in the insoluble fractions, the soluble fraction from a 100-day-old non-Tg mouse was used as a positive control. β-Actin in whole homogenates was used as a loading control. (**B**, **C**) Scatter plots showing the relative expression levels of insoluble (**B**) total and (**C**) soluble murine SOD1 proteins. ***P*<0.01 (vs. the disease stage-matched G85R).
