# Supplementary material for: Diverse effects of coexpression of human SOD1 variants on motor neuron disease
Source: Hum Mol Genet. 2025 Jun 1;34(16):1380–91. doi: 10.1093/hmg/ddaf088 (PMC12361113; doi:10.1093/hmg/ddaf088)
Supplement: Supplementary_Table_S5_ddaf088 [file supplementary_table_s5_ddaf088.docx]

|  | G85R  (n = 3) | G85R/D90A  (n = 3) | G85R/WT  (n = 3) | WT/WT  (n = 2) |
| --- | --- | --- | --- | --- |
| aa 86-94 (WT) | 4.1 ± 0.6 | 1.7 ± 1.0 | 2.8 ± 0.5 | 6.6, 6.6 |
| aa 86-94 (D90A) | 0 | 2.6 ± 2.5 | 0 | 0, 0 |

**Supplementary Table S5 Epitope mapping of aggregates in Tg mice of various combinations.**

The antibodies were raised against peptides corresponding to the 86–94 sequences in hSOD1^WT^ and hSOD^D90A^. Three mice of each genotype were analyzed, except for the homozygous hSOD1^WT/WT^ mice from which only two samples were available. The figures indicate the reactivities of the samples with the aa 86-94 antibodies as a percentage of the reactivities with the aa 57–72 antibody (mean ± S.D.). A pool of five samples from non-Tg C57BL/6J mice was used as a blank. 0 indicates reactions with the mutant-specific antibodies that were equal to or lower than that of the blank.
